# Simulation of sugar kelp (*Saccharina latissima*) breeding guided by practices to accelerate genetic gains

**DOI:** 10.1093/g3journal/jkac003

**Published:** 2022-01-19

**Authors:** Mao Huang, Kelly R Robbins, Yaoguang Li, Schery Umanzor, Michael Marty-Rivera, David Bailey, Charles Yarish, Scott Lindell, Jean-Luc Jannink

**Affiliations:** 1 Section on Plant Breeding and Genetics, School of Integrative Plant Sciences, Cornell University, Ithaca, NY 14853, USA; 2 Department of Ecology & Evolutionary Biology, University of Connecticut, Stamford, CT 06901-2315, USA; 3 College of Fisheries and Ocean Sciences, University of Alaska Fairbanks, Juneau, AK 99775, USA; 4 Applied Ocean Physics and Engineering Department, Woods Hole Oceanographic Institution, Woods Hole, MA 02543, USA; 5 United States Department of Agriculture—Agriculture Research Service, Ithaca, NY 14853, USA

**Keywords:** sugar kelp, *Saccharina latissima*, simulation, breeding, genetic gain, genomic selection, Genomic Prediction, GenPred, Shared Data Resource

## Abstract

Though *Saccharina japonica* cultivation has been established for many decades in East Asian countries, the domestication process of sugar kelp (*Saccharina latissima*) in the Northeast United States is still at its infancy. In this study, by using data from our breeding experience, we will demonstrate how obstacles for accelerated genetic gain can be assessed using simulation approaches that inform resource allocation decisions. Thus far, we have used 140 wild sporophytes that were sampled in 2018 from the northern Gulf of Maine to southern New England. From these sporophytes, we sampled gametophytes and made and evaluated over 600 progeny sporophytes from crosses among the gametophytes in 2019 and 2020. The biphasic life cycle of kelp gives a great advantage in selective breeding as we can potentially select both on the sporophytes and gametophytes. However, several obstacles exist, such as the amount of time it takes to complete a breeding cycle, the number of gametophytes that can be maintained in the laboratory, and whether positive selection can be conducted on farm-tested sporophytes. Using the Gulf of Maine population characteristics for heritability and effective population size, we simulated a founder population of 1,000 individuals and evaluated the impact of overcoming these obstacles on rate of genetic gain. Our results showed that key factors to improve current genetic gain rely mainly on our ability to induce reproduction of the best farm-tested sporophytes, and to accelerate the clonal vegetative growth of released gametophytes so that enough gametophyte biomass is ready for making crosses by the next growing season. Overcoming these challenges could improve rates of genetic gain more than 2-fold. Future research should focus on conditions favorable for inducing spring reproduction, and on increasing the amount of gametophyte tissue available in time to make fall crosses in the same year.

## Introduction

Wild kelp forests in the ocean provide important habitat and ecosystem services. They have also been an important source of human food. Due to climate change and other anthropogenic factors, global kelp populations have faced a drastic decline (Moy and Christie 2012; Wernberg *et al.* 2019; Bricknell *et al.* 2021). Now kelp farming is largely replacing wild harvests: over 32 million metric tons of seaweed were harvested in 2020, of which 97% came from farms (FAO 2020). The import of seaweed raw materials to the United States in 2016 was more than 10,000 metric tons (over $73 million; National Marine Fisheries Service Office of Science and Technology 2016; Piconi *et al.* 2020). Uses include human food, animal feed supplements, and pharmaceutical and cosmetic products (Kim *et al.*^2015^, ^2017^, ^2019^; Marine Biotech 2015; Schiener *et al.* 2015; Yarish *et al.* 2017; Wang *et al.* 2020). Growing kelp biomass in the ocean offers a unique opportunity to avoid many of the challenges associated with terrestrial agriculture systems, particularly the growing competition for arable land and freshwater resources. In order to meet the demand of our growing population by 2050, we must use the oceans responsibly to build a thriving seaweed farming industry for the production of carbon-neutral fuels, biochemicals, animal feed, and food (Capron *et al.* 2020; Vijn *et al.* 2020).

Kelp cultivation has been established for over 60 years in Asian countries. More recently, there is growing interest in macroalgal cultivation in Europe, South America, and North America (Buschmann *et al.* 2017; Grebe *et al.* 2019; Kim *et al.* 2019; Goecke *et al.* 2020). Specifically, there are efforts to selectively breed kelp for large-scale food and bioenergy production (Bjerregaard *et al.* 2016; Valero *et al.* 2017; Hwang *et al.* 2019; Goecke *et al.* 2020) as well as increased demand for germplasm banking to support future cultivation (Barrento *et al.* 2016; Wade *et al.* 2020; Yang *et al.* 2021). The US Department of Energy Advanced Research Projects Agency-Energy (ARPA-E) initiated the Macroalgae Research Inspiring Novel Energy Resources (MARINER) program to develop new cultivation, management, and breeding technologies that enable cost-efficient seaweed farming in the large US Exclusive Economic Zone and grow the United States into a global leader in the production of seaweeds. *Saccharina japonica* breeding strategies that have been used in the past involved selfing, which leads to loss of genetic diversity (Hu *et al.* 2021). In contrast, in this project, we take advantage of genomic tools newly developed in *Saccharina latissima* and apply genomic selection (GS), with the aim of improving breeding efficiency and predicting combining abilities of parental gametophytes (GPs) using the sporophyte (SP) performance. As the first breeding program for sugar kelp in the Northeast United States, we initiated a selective breeding system by evaluating uniclonal plots.

**Fig. 1.**
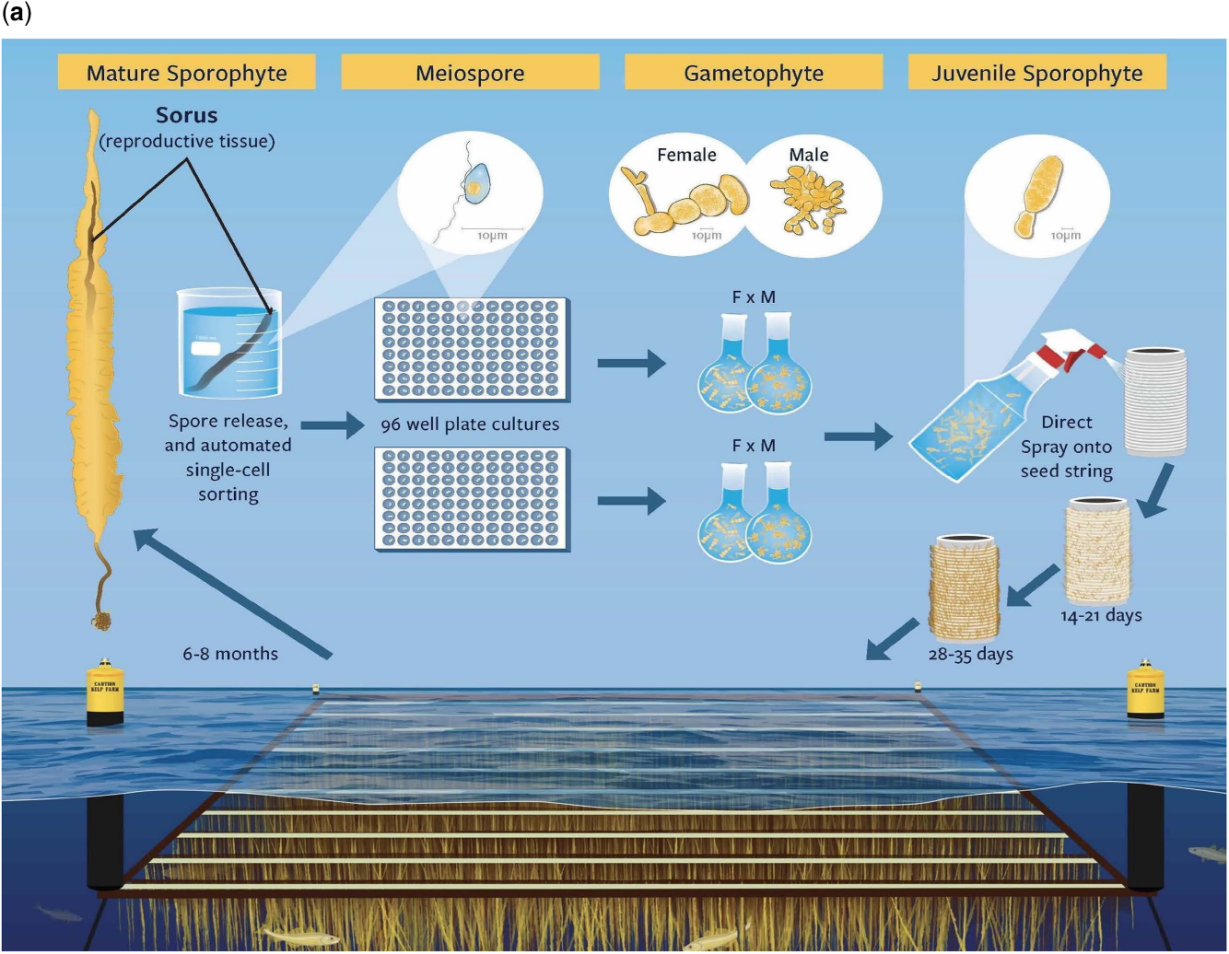

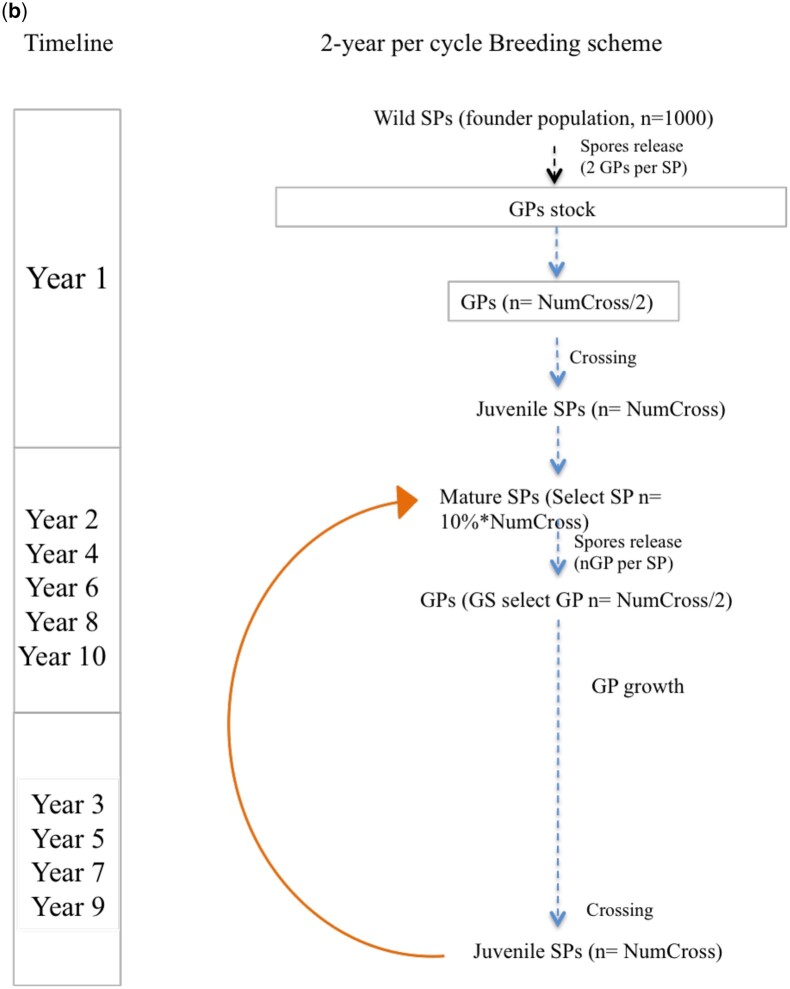

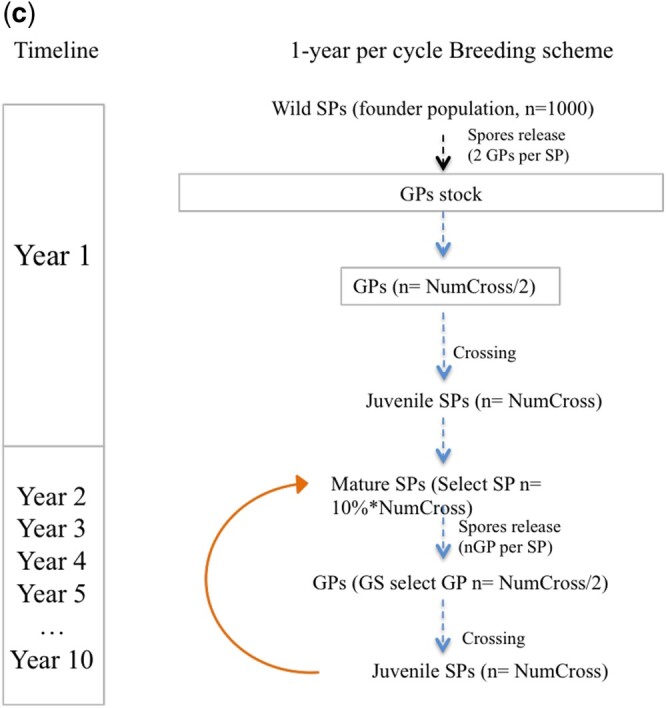
a) Biphasic life cycle and breeding pipeline of sugar kelp (*S. latissima*) in our research project. Represented are meiospore release, flow cell sorting to 96-well plates, propagation to sufficient biomass for crossing, spraying of crossed SPs onto seed string, and outplanting to a farm-like common garden field experiment. b) Simulated breeding scheme with a 2-year breeding cycle, where nGP refers to either 24 or 96 GPs generated per SP; NumCross corresponds to making and evaluating 400 versus 1,000 crosses; SelectSP corresponds to either random or phenotypically selecting top performing SPs. The SPs were genotyped and phenotyped and GPs were genotyped. GS model (ridge regression BLUPs) was built using SPs data to predict GEBVs of GPs, and GPs were selected based on their GEBVs. c) Simulated breeding scheme of 1-year per breeding cycle. Parameter abbreviations are the same as in Fig. 1b.

Genetic markers have been used in crop breeding for some time, primarily exploiting large marker-trait associations (Bernardo 2016). In the last decade, GS has been adapted by numerous breeding programs due to its ability to predict breeding values that are immediately used for making selections (Meuwissen *et al.* 2001; Jannink *et al.* 2010). The use of GS in terrestrial agriculture and aquaculture breeding has a track record of improving gains by ∼10% per generation (Gjedrem *et al.* 2012). GS uses a training population with both phenotypic and genotypic information to build a model, which then can be used to predict the genomic estimated breeding value (GEBV) of individuals that are related to the training population. As the development of genetic markers and genotyping individuals becomes less costly compared with phenotyping, GS allows breeders to make selections more efficiently, especially in early years’ breeding evaluations (Heffner *et al.* 2010; Borrenpohl *et al.* 2020). Genetic markers have been used for marker assisted breeding in seaweed (Hu *et al.* 2021), however, there are no reports about applying GS in sugar kelp breeding. Kelp has a biphasic life cycle (Redmond *et al.* 2014; Fig. 1a), which provides unique opportunities for selective breeding since breeders could potentially exert selection pressure on both phases within a single growing cycle (Peteiro *et al.* 2016; Wade *et al.* 2020). The use of GS on the GP phase will enable us to prioritize crosses and evaluate SPs that are more likely to become high-performing varieties.

We initiated a sugar kelp breeding program in 2018, and our primary breeding goal is to improve biomass-related traits including wet weight and ash free percentage dry weight, and to reduce biomass ash content. With 3 years of breeding experience, we have identified limiting factors to our breeding effort and the investments that might be exerted to overcome those limitations (Umanzor *et al.* 2021). To guide the research effort objectively, the extent of accelerated gain from different possible investments and interventions needs to be assessed via simulation. These simulations will help early kelp breeding efforts utilize limited research and development investment for maximal breeding efficiency and genetic gain (the improvement of the population genetic mean). Simulation studies have been a useful tool in assisting breeders’ decision-making. They are often used to dissect problems that are difficult (expensive or time consuming) to address experimentally. Simulation models can be used to refine crop management strategies based on prior results and experience. Examples include exploring different ways to improve nitrogen use efficiency for wheat (Dresbøll and Thorup-Kristensen 2014), identifying the best field experimental designs to control for spatial variation in agriculture and forestry studies (Gezan *et al.* 2010), and assessing potential genetic gains in a small young sorghum breeding program (Muleta *et al.* 2019). Simulation incorporating GS has been applied in terrestrial plants, such as barley (*Hordeum vulgare* L. Iwata and Jannink 2011) and *Cryptomeria japonica* (Iwata *et al.* 2011). Comparisons of GS vs phenotypic selection were evaluated in simulation so that breeders could strategically allocate resources between genotyping vs phenotyping (Hickey *et al.* 2014), and between the sizes of populations vs numbers of replications to be tested (Lorenz 2013). In aquaculture, breeding simulation studies have also been applied to address a variety of questions (Zenger *et al.* 2019), including assessing the changes of inbreeding rates over time (Bentsen and Olesen 2002), evaluating the effects of mating strategies on the changes of genetic gain in 10 generations of aquaculture selection (Sonesson and Ødegård 2016), and assessing the genomic prediction accuracy using either identical by state or identical by descent genomic relationship matrices (Vela-Avitúa *et al.* 2015). Zenger *et al.* (2019) reported that at least 36 simulation studies were relevant in aquaculture breeding evaluating different mating designs, selection strategies, family and genome sizes, and their effects on changes of breeding program over different generations. To the best of our knowledge, there have been no simulation studies on breeding schemes applied to sugar kelp and we propose the first one.

For simulation studies to be valuable guides, they must be appropriately parameterized. Our simulation parameters were chosen on the basis of 2 different levels of both trait heritability, as estimated from nearshore farm plot data, and effective population size, as estimated by using marker linkage disequilibrium (LD) among founders. The objective of this study was to determine the impacts of overcoming these 4 obstacles on genetic gain over 10 years of breeding effort. These obstacles are further explained in *Materials and Methods*.

## Materials and methods

### Sugar kelp breeding program initiation

In 2018, we collected SPs from the wild throughout the Gulf of Maine (GOM) to southern New England (SNE) in the United States (Mao *et al.* 2020). Population genetic analyses on the wild samples were performed to understand their diversity, the relationships among them, and their population history in terms of effective population size (Mao *et al.* 2020).

From the wild-sampled SPs founders, over 700 uniclonal GPs were isolated and grown to sufficient biomass for genotyping and for crossing to create progeny SPs. These SPs were then planted and evaluated on nearshore kelp farms with an augmented design, where only check crosses were repeated across blocks. Given that SNE formed a distinct subpopulation structure from GOM (Mao *et al.* 2020), and we lacked phenotypic data from SNE sites, all simulation parameters used in this study were based on the GOM population. There were 2 checks and 9 blocks in the 2019 planting season and 3 checks and 11 blocks in the 2020 planting season. Within a cross, each SP has the same genotype, resulting in genetically uniform 1-m line “plots” in the farm. In our breeding work, we have measured various traits at plot and individual blade levels (Umanzor *et al.* 2021). At harvest time for the 2019 and 2020 seasons, the farm-grown SPs were phenotyped and sampled. The mature sorus tissue from SPs were collected to induce GPs in the laboratory for the next crossing, planting, and harvesting cycle. In our current scheme, we use GS to predict the breeding value of GPs, select the best ones, and prioritize crossing the best GPs to create new SPs.

We considered the year collecting wild samples in the breeding program as a burn-in period (for which time is needed to culture enough clonal biomass for breeding), and our repeated breeding cycles start from year 1, for both the 2-year cycle and 1-year cycle breeding schemes (Fig. 1b). A full breeding cycle starts from farm-grown mature SPs, from which we collect spores and generate GPs. Then the GPs are clonally propagated and crossed, and a new generation of mature SPs is evaluated on farm (Fig. 1b). For simplification, and given that our goal is to compare genetic gain change due to different obstacles, we did not simulate overlapping breeding cycles where GPs from historical cycles would be used in a current cycle, but focused on nonoverlapping cycles where GPs used for crossing always come from the same cycle (Fig. 1b).

### Defining the 4 major obstacles

Thus far, we have identified 4 limiting factors (obstacles) in the breeding program (Table 1). First, *Obstacle 1* (CycleTime, Table 1) refers to the amount of time it requires to complete a breeding cycle. CycleTime is dependent on the growth of GPs during clonal propagation from a single meiospore. Due to the slow growth, it has thus far not been possible to accumulate enough GP biomass to cross in the same calendar year that the meiospore was obtained.

**Table 1.** Definition of simulation obstacles and the corresponding parameters.

Obstacles	Simulation parameters	Definition	Related factors	Reference scheme parameters	Changed scheme parameters
Obstacle 1	CycleTime	Breeding cycle time	Slow growth of GP	2	1
Obstacle 2	NumCross	Number of crosses possibly made and tested	Labor intensity to maintain large number of GP cultures	400	1,000
Obstacle 3	SelectSP	Selection on SP	SPs do not become reproductive	Random selecting 10%	Phenotypic selecting top 10%
Obstacle 4	nGP	Number of GPs isolated from each SP	GPs survival varies through flow cytometry process	24	96

The technical advancement to overcome *Obstacle 1* and complete a breeding cycle in 1 year would entail some combination of the following:

1. Methods to enhance the growth rate of the GPs so that GPs sampled in the spring have sufficient biomass to make crosses in the fall.
2. Methods to make crosses that require less GP biomass but that nevertheless produce plots with adequate numbers of SPs.

In our empirical breeding program, we worked with CycleTime = 2 years. We simulated scenarios where we have a 2-year cycle time and where we could reduce the time from 2 years to 1 year.

*Obstacle 2* (NumCross, Table 1) refers to the number of SP plots being evaluated on a farm. This obstacle is related to limited capacity to grow GPs for crossing. Limiting the number of crosses and associated phenotypic variance can reduce the expected selection intensity and genetic gain. Overcoming *Obstacle 2* would require the ability to maintain and culture more GPs in limited laboratory space with more cost and labor effective methods.

Currently, we are limited to making no more than 400 crosses per year in our empirical breeding program, due to the labor intensity of maintaining and growing individual GP cultures (all year around), and seasonal capacity in the hatchery for maintaining GP crosses and the resultant individuals of uniclonal juvenile SPs for 4–6 weeks before out-planting. We simulated scenarios assuming we could design space and labor-saving methods for the laboratory/hatchery phase and manage higher throughput phenotyping to evaluate NumCross = 1,000 plots instead of NumCross = 400 plots each year.

*Obstacle 3* (SelectSP, Table 1) refers to selecting SPs either randomly or phenotypically. In our empirical program, we have had minimal ability to exert positive selection on farm-tested SPs using their phenotypic data because we have not able to induce many top-ranked SPs to become reproductive. Thus, the next generation of GPs was essentially obtained from randomly picked SPs (SelectSP = random, Table 1). Overcoming *Obstacle 3* would entail rapid identification of top SPs, and artificial laboratory induction of SPs to enter their reproductive phase with high rate of spore release (Pang and Lüning 2004). We simulated scenarios where all the top-ranked SPs could be artificially manipulated to be reproductive (Select SP = pheno, Table 1), hence we could perform phenotypic selection on these SPs rather than applying random selection (Pang and Lüning 2004).

Finally, *Obstacle 4* (nGP, Table 1) refers to the GP survival rate per 96-well plate, which relates to the number of GPs we collect per parental SP. We have isolated meiospores individually into 96-well plates using flow cytometry (Augyte *et al.* 2020). This automated spore sorting technology produces viable uniclonal isolations from spores released by each individual SP. This sorting method showed a maximum effectiveness of 76% in GP development (Augyte *et al.* 2020). Investment in the flow cytometry method to either increase GP survival or enable the preparation of more plates, thus generating more GPs from which to select, would overcome this 4th obstacle.

We estimated the average GP survival per plate as nGP = 24 from our current breeding program (Table 1). Hence 24 is our reference parameter, whereas an ideal situation where all GPs in the plate are viable (nGP = 96, Table 1) was simulated. Generating nGP = 96 GPs means there will be 4 times more GPs than nGP = 24 to select from to make crosses for SPs, enabling higher selection intensity.

Breeding program settings of CycleTime = 2 years, NumCross = 400 plots, SelectSP = Random, and nGP = 24 resemble our current empirical program and were considered as our baseline. Our main goal was to determine which obstacles most hinder genetic gain, such that overcoming them leads to the greatest response. Hence each of these obstacles was considered as a factor in our analysis.

### Founder population effective population size

To simulate breeding schemes overcoming these obstacles, we first needed an estimate of the effective population size of the kelp founder population. We used marker data on 125 wild SPs, sampled from the GOM and genotyped via DArT technology (Mao *et al.* 2020) to obtain this estimation. Data cleaning was similar to Mao *et al.* (2020). Markers were filtered by removing ones with more than 10% missing data and those severely departing from Hardy–Weinberg Equilibrium (*P*-value < 0.01) in more than 25% of the collection sites. Markers with minor allele frequency <0.05 and individuals with more than 50% missing data were also removed. A final set of 4,906 markers was retained and imputed using the rrBLUP package A.mat function (Endelman 2011) in R (R Development Core Team 2018). LD between markers was estimated using the genetics package (Warnes *et al.* 2012). The average LD score was estimated to be 0.08, which was then used in estimating the effective sample size (*N*_e_), according to Sved (1971):

$$E\left(r^{2}\right)\approx \frac{1}{1+4N_{e}c},$$

where $E\left(r^{2}\right)$ is the expected $r^{2}$ for which we used the average LD score of 0.08, and *c* is the recombination rate among all sites assumed to be 0.5, given that most pairs of sites are on different chromosomes. This gives an estimated $N_{e}=60$. We know that the GOM population is strongly structured (Mao *et al.* 2020), which may cause *N*_e_ to be underestimated (Lande and Barrowclough 1987). Thus, we also ran simulations with a setting of $N_{e}=600$. A total of 1,000 SP individuals were simulated as our founders with an effective population size of either $N_{e}=60$ or $N_{e}=600$.

### Trait heritability

We used empirical data to estimate trait heritability for the simulation. A mixed model, including genetic effects of SPs as random effects, and growth line, blocks, year effects, and reference checks as the fixed effects, was conducted to estimate the narrow sense heritability using GOM farm SP plots data from 2019 and 2020 field seasons. The following model was used:

$$Y_{\mathrm{ijkln}}=\;µ+E_{i}+C_{j}+B_{k}(E_{i})+L_{l}(E_{i})+G_{n}(C_{j})+e_{i}$$

$Y_{\mathrm{ijkln}}$ is the *ijkln*th observation, *µ* is the mean, $E_{i}$ is the *i*th environment (year), $C_{j}$ is the *j*th check groups, where each check variety is a unique group whereas all testing crosses were assigned as 1 combined group, $B_{k}(E_{i})$ and $L_{l}(E_{i})$ is the *k*th block and *l*th line effects nested within year, respectively, $G_{n}(C_{j})$ is the genetic effects for the *n*th individual (uniclonal SP plot) nested within *j*th check group. All effects were treated as fixed except for SP genetic effects. A relationship matrix was included using the rrBLUP mixed.solve() function in R to obtain the additive variance for SPs. Any interaction between genotype and environmental variation from year to year (such as water temperature, light, etc.) would be part of the error variance. Narrow sense heritability was estimated using:

$$h^{2}=\frac{\sigma_{A}^{2}}{\sigma_{A}^{2}+\sigma_{E}^{2}},$$

where $\sigma_{A}^{2}$ was the estimated additive variance for the SPs and $\sigma_{E}^{2}$ was the error variance from a mixed model. Trait heritabilities ranged from 0.06 to 0.43 for plot-level traits and 0.59–0.82 for blade-level traits using both years’ data. We choose heritabilities of 0.20 and 0.50 in the simulation to cover plausible values for the biomass trait. We set the trait genotypic variance to 1, resulting in error variances of 4 and 1, for the respective heritabilities.

### Breeding pipeline in AlphaSimR

Breeding scheme simulations were performed using functions in the AlphaSimR package (Gaynor *et al.* 2021). We simulated initial SP founder populations of 1,000 individuals using the following parameters (Fig. 1, b and c; Table 1): The ploidy level was set to 2 and the number of chromosomes was assumed to be 31 based on the close congener *S.japonica* (Liu *et al.* 2012). Per chromosome, the number of segregating sites and the number of QTL were set to 500 and 100, respectively. The positions of all sites were random over a recombination length of 1 Morgan of each of the 31 chromosomes. AlphaSimR simulated downstream recombination using the gamma model (McPeek and Speed 1995) parameterized to approximate the Kosambi mapping function. These values assume that the trait is polygenic but are otherwise somewhat arbitrary and chosen referring to those in Muleta *et al.* (2019). Historically, we have been able to produce enough GP biomass to make 2 crosses per GP. Consequently, we assumed that same capacity in the simulation scheme, and allowed each founder SP to generate 2 GPs, giving enough GPs to make NumCross (Table 1; Fig. 1b) crosses for downstream SP generations without exerting selection pressure on the founder population. The simulation program randomly assigned “F” and “M” sexes to GPs generated from the SP populations (Fig. 1b). In our simulation, among the selected GP parents, the mating scheme is random, which means it is possible for a female GP and male GP from the same SP to be crossed with each other (equivalent to selfing). Since we simulated additive gene action, occasional self should not be an issue. We chose an additive model because we have no evidence for either heterosis or inbreeding depression in our breeding program thus far. Ten percentage of SPs were selected either randomly or based on phenotypes and then used to produce the next generation GPs. Thus, 40 (when NumCross = 400) or 100 SPs (when NumCross = 1,000) were selected at the SP selection stage, respectively (Fig. 1, b and c). We then assumed flow cytometry would be used to obtain GPs from each selected SP (Augyte *et al.* 2020).

Phenotypes were simulated for SPs using *setPheno()* function in AlphaSimR, which calculates genotypic values based on an individual’s genotype and then adds a random error deviation. We specified 2 different values for error variance based on the heritabilities of 0.2 and 0.5. The farm-evaluated SPs and their genotypic and phenotypic data from all previous breeding cycles were used to train a GS model using the *RRBLUP()* function. Hence, the GS training population size increased over time and was updated each new generation by feeding in new SP phenotypic data. The GS training size at the *i*th year (*i* > 2) is NumCross*(*i* − 1), and the end training size by Year 10 is NumCross*9. All GPs coming out of the flow cytometry process would be genotyped. The GS model used ridge regression best linear unbiased prediction (Meuwissen *et al.* 2001), and the GEBVs were obtained with the *setEBV()* function. GS accuracy was defined as the correlation of estimated breeding values and the true breeding values, extracted via the *ebv()* and *gv()* functions in AlphaSimR, respectively. Each GP can make 2 crosses, and hence *n* = NumCross/2 GPs of each sex would be selected based on their GEBVs in order to generate NumCross SP plots to evaluate on farm (Fig 1, b and c; Table 1). Note that with this scheme, changing the number of SPs crossed and evaluated does not change the selection intensity either during SP or GP selection, whereas changing the number of GPs generated changes the selection intensity during the GP selection stage. These simulation procedures were used on a full factorial of nGP (24 vs 96) × heritability (0.2 vs 0.5) × Ne (60 vs 600) × NumCross (400 vs 1,000) × SelectSP (random vs pheno) × CycleTime (2 years vs 1 year) for a total of 64 simulation settings.

### Estimating genetic gain, genetic variance, and GS accuracy over 10 breeding years

Each scheme was simulated 20 times, and the average genetic gain as well as genetic variance at each GP stage was calculated over 10 years. Because we were mainly interested in evaluating the trend of genetic gain from different breeding schemes, the reference point for genetic gain could either be for GPs or SPs and we used the GP stage. The additive genetic variation, selection accuracy, selection intensity, and breeding cycle time can affect genetic gain:

$$\Delta G=\frac{\sigma_{a}ir}{L},$$

where ΔG is the genetic gain, $\sigma_{a}$ is the additive genetic variation, *i* is the selection intensity, *r* is the selection accuracy, and *L* is the cycle time (Lush 1937; Eberhart 1970). The trait we simulated was a general yield related trait with arbitrary units. We compared effects caused by the changes of parameters on genetic mean value (estimated via the function *meanG* in AlphaSimR), total genetic variance (estimated via the function *varG* in AlphaSimR), and GS prediction accuracy via ANOVA.

### Selection intensity

Our number of GPs per SP (nGP) was either 24 or 96. At any given generation of SPs, 10% of lines were selected either at random or by phenotypically selecting the top performers. The selection intensity under random selection is 0, and selecting the top 10% assuming a normal distribution causes the selection intensity to be 1.75. When selecting among GPs, with a scenario of nGP = 24, the proportion of FG and MG being selected is 41.6% [NumCross/(10%*NumCross*24)]. Again assuming normality, the selection intensity is 0.94. When nGP = 96, the proportion being selected is 10.4% [NumCross/(10%*NumCross*96)], generating a selection intensity of 1.74 (again assuming normality). To validate the normality assumption, we evaluated empirical selection intensity by calculating the standardized mean difference between the selected population and the reference population. These empirical intensities were similar to the theoretical values (data not shown).

### Analysis of variance

The analysis of variance (ANOVA) was conducted to evaluate effects of SelectSP, NumCross, CycleTime, and nGP as well as their interaction effects on changes of genetic mean, genetic variance, and GS accuracy, respectively, over 10 years of breeding time. The ANOVA was reported at each of the Ne and *h*^2^ levels.

## Results

### Simulation output

The ability to exert selection on the farm-evaluated SPs (SelectSP), the number of years per breeding cycle (CycleTime), and the number of GPs per SP surviving the flow cell cytometry system (nGP) were the 3 significant contributors to the changes of genetic mean over time (Table 1). We did not observe significant interactions between these factors (Table 2).

**Table 2.** ANOVA on genetic mean split by founder effective population size (*N*_e_) and heritability (*h*^2^).

	Df	Sum Sq	Mean Sq	*F*	*P*-value
(a) *N*_e_ = 60, *h*^2^ = 0.5					
SelectSP	1	81.7	81.7	20.5	0.000^c^
NumCross	1	3.8	3.8	0.9	0.334
CycleTime	1	62.2	62.2	15.6	0.000^c^
nGP	1	28.5	28.5	7.2	0.009^b^
SelectSP:NumCross	1	0.1	0.1	0.0	0.866
SelectSP:CycleTime	1	1.5	1.5	0.4	0.539
SelectSP:nGP	1	1.7	1.7	0.4	0.519
NumCross:CycleTime	1	0.2	0.2	0.1	0.809
NumCross:nGP	1	0.4	0.4	0.1	0.764
CycleTime:nGP	1	1.5	1.5	0.4	0.543
Residuals	101	402.6	4.0		
(b) *N*_e_ = 600, *h*^2^ = 0.5					
SelectSP	1	85.6	85.6	31.1	0.000^c^
NumCross	1	1.1	1.1	0.4	0.529
CycleTime	1	43.4	43.4	15.8	0.000^c^
nGP	1	15.3	15.3	5.6	0.020^a^
SelectSP:NumCross	1	0.5	0.5	0.2	0.676
SelectSP:CycleTime	1	1.3	1.3	0.5	0.497
SelectSP:nGP	1	1.7	1.7	0.6	0.439
NumCross:CycleTime	1	0.1	0.1	0.0	0.851
NumCross:nGP	1	0.7	0.7	0.2	0.627
CycleTime:nGP	1	0.5	0.5	0.2	0.668
Residuals	101	278.2	2.8		
(c) *N*_e_ = 60, *h*^2^ = 0.2					
SelectSP	1	35.7	35.7	15.2	0.000^c^
NumCross	1	5.1	5.1	2.2	0.145
CycleTime	1	36.8	36.8	15.6	0.000^c^
nGP	1	17.8	17.8	7.5	0.007^b^
SelectSP:NumCross	1	0.1	0.1	0.0	0.832
SelectSP:CycleTime	1	0.5	0.5	0.2	0.630
SelectSP:nGP	1	0.5	0.5	0.2	0.640
NumCross:CycleTime	1	0.1	0.1	0.0	0.831
NumCross:nGP	1	0.7	0.7	0.3	0.587
CycleTime:nGP	1	1.0	1.0	0.4	0.515
Residuals	101	237.6	2.4		
(d) *N*_e_ = 600, *h*^2^ = 0.2					
SelectSP	1	34.4	34.4	21.8	0.000^c^
NumCross	1	1.4	1.4	0.9	0.345
CycleTime	1	24.0	24.0	15.2	0.000^c^
nGP	1	8.1	8.1	5.1	0.026^a^
SelectSP:NumCross	1	0.4	0.4	0.3	0.613
SelectSP:CycleTime	1	0.5	0.5	0.3	0.587
SelectSP:nGP	1	0.6	0.6	0.4	0.546
NumCross:CycleTime	1	0.2	0.2	0.1	0.701
NumCross:nGP	1	1.1	1.1	0.7	0.401
CycleTime:nGP	1	0.2	0.2	0.1	0.704
Residuals	101	159.3	1.6		

SelectSP, selection among SP based on phenotype or at random; NumCross, common garden of 400 versus 1,000 field plots; CycleTime, 1-year versus 2-year cycle; nGP, number of GPs obtained per parental SP of 24 or 96.

a *P* < 0.05.

b *P* < 0.001.

c *P* < 0.0001.

### Genetic mean

**Fig. 2.**
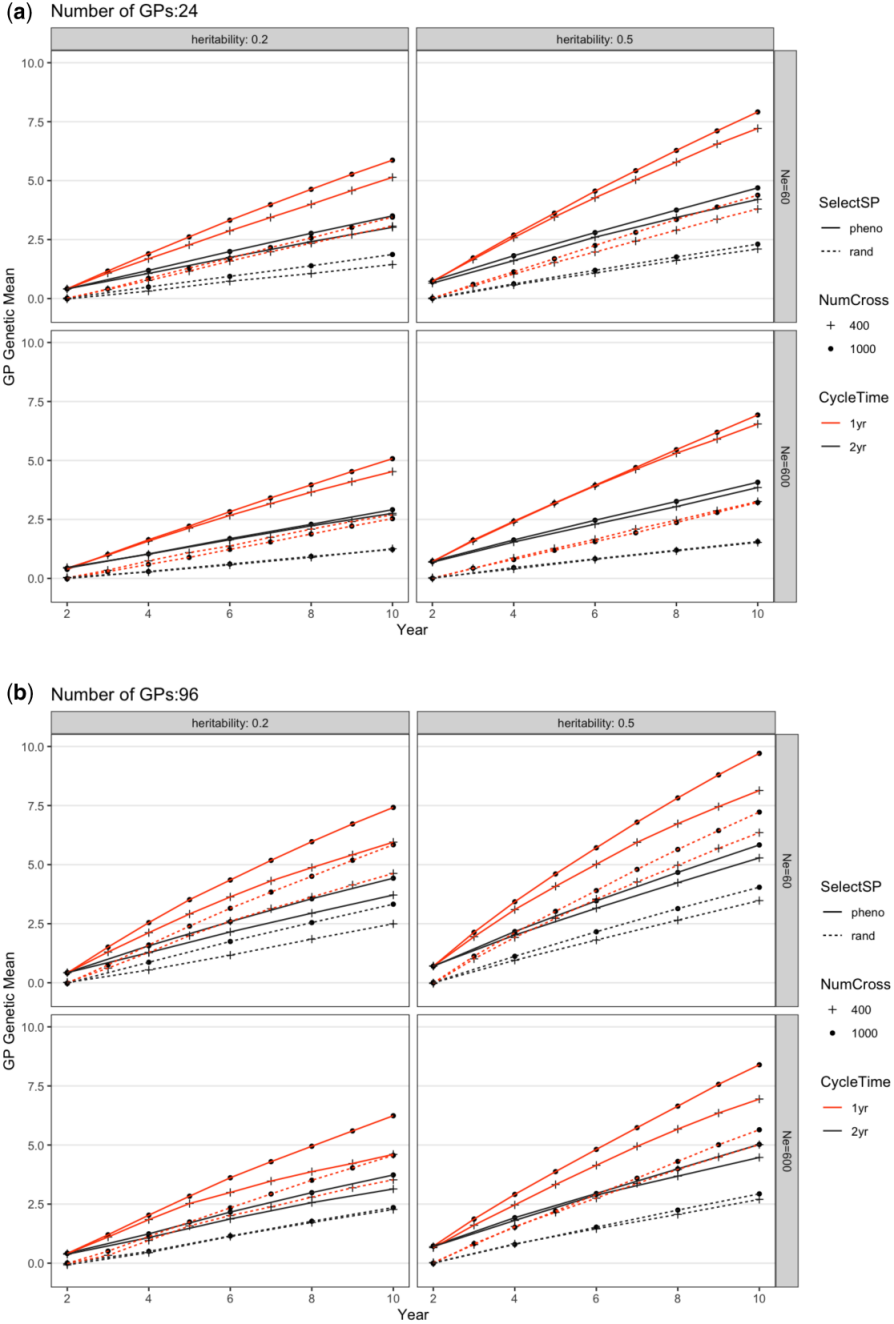
Genetic mean of the GP population from different breeding schemes over 10 years. The routine breeding scheme starts in year 2. Each figure shows SelectSP: selecting the best (pheno) vs random (rand) SPs; NumCross: Evaluating 400 vs 1,000 crosses; and CycleTime: 1-year (1 yr) vs 2-year (2 yr). Subpanels separate different founder population effective population sizes of 60 (Ne60) and 600 (Ne600) and trait heritabilities of *h*^2^=0.5 and *h*^2^=0.2 when a) 24 or b) 96 GPs were propagated from each parental SP. Each scheme was repeated 20 times and genetic values shown were averages. The SE was smaller than the figure symbols and is not shown. The trait simulated is an arbitrary economic value trait with an initial variance of 1.

The changes from Fig. 2, a to b reflect the effects of overcoming *Obstacle 4* (nGP = 24 vs nGP = 96, Tables 1 and 2). This change led to a gain increase of 35% averaged across all other factors (nGP, Table 2; Fig. 2). Relative to the baseline, the ability to exert selection on SPs (SelectSP, *Obstacle 3*) and decreasing the breeding cycle time (CycleTime, *Obstacle 1*) led to gain increases of 118% and 73%, respectively, averaged across all other factors. Though the effect of increasing the number of plots phenotyped (NumCross, *Obstacle 2*) was not statistically significant (Tables 1 and 2), numerically this change increased gain by an average of 7%. We did not observe significant interactions: the effects of overcoming each obstacle were additive (Table 2), and overcoming all 4 obstacles led to the greatest gain (nGP = 96, SelectSP = phenotypic, CycleTime = 1 year, and NumCross = 1,000; Fig. 2). Heritability also played a role in affecting the genetic gain (Table 2), where *h*^2^ = 0.5 generated higher genetic mean after 10 years of breeding than *h*^2^ = 0.2 (Fig. 2, a and b). This trend was consistent regardless of the number of GPs or effective population size.

### Genetic variance

**Fig. 3.**
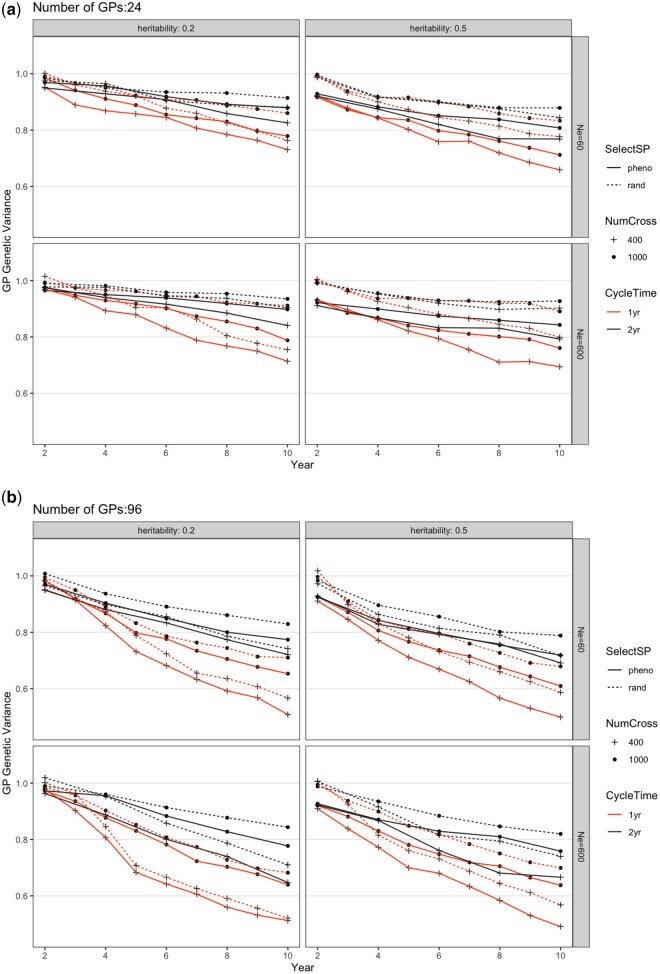
Change of total genetic variance in the GP population from different breeding schemes over 10 years for a) 24 or b) 96 GPs per parental SP. The scheme abbreviations are the same as in Fig. 2. Each scheme was repeated 20 times and genetic variance shown was the average. The SE was smaller than the figure symbols and is not shown. The trait simulated is an arbitrary economic value trait with an initial variance of 1.

The breeding scheme interventions simulated also affected the genetic variance remaining after 10 years of improvement (Fig. 3). As expected, total genetic variance decreased over time. The 4 factors (SelectSP, NumCross, CycleTime, and nGP) all had significant effects on the changes of genetic variance over 10 years (Supplementary Table 1; Fig. 3). Compared with the baseline, genetic variance decreased 6.9% and 4.7% as a result of selecting SPs on phenotype (SelectSP) and changing CycleTime from 2 years to 1 year, respectively, whereas it *increased* 4.0% by increasing NumCross from 400 to 1,000 plots. Overall, changing from nGP = 24 to nGP = 96 decreased genetic variance by 9.2% over 10 years. There was a low level of interactions between simulated factors (Supplementary Table 1), which can also be seen in Supplementary Fig. 1 by the fact that lines linking simulation settings with and without the interventions are approximately parallel and of similar length, indicating that changing 1 factor has basically the same effect regardless of the levels of the other factors.

### GS accuracy

**Fig. 4.**
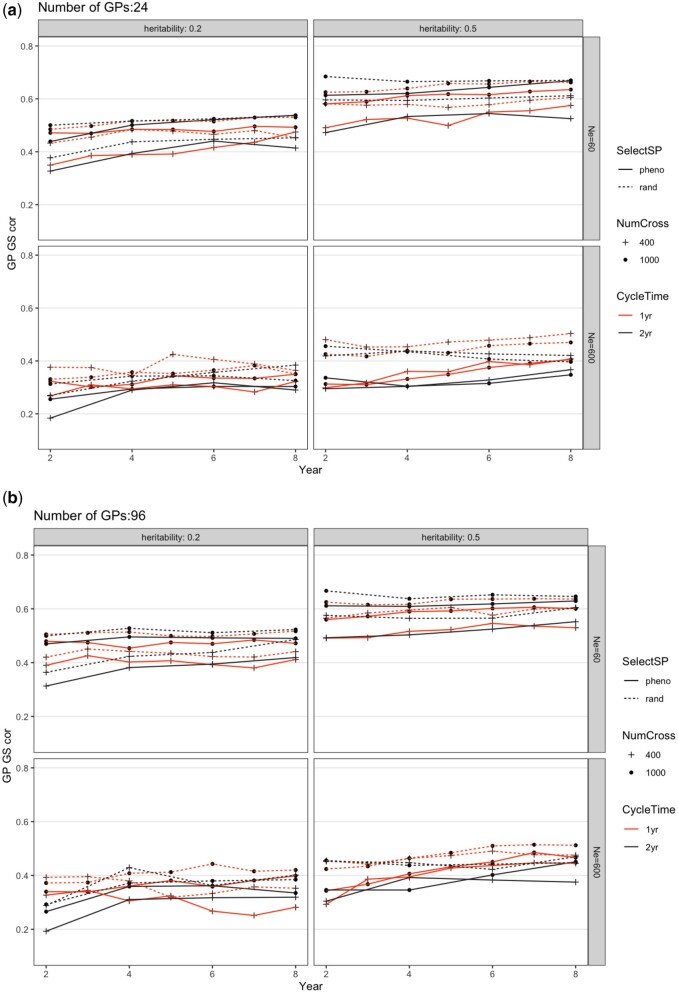
Change of genomic prediction accuracy in the GP population from different breeding schemes over 10 years for a) 24 or b) 96 GPs per parental SP. The scheme abbreviations are the same as in Fig. 2. Each scheme was repeated 20 times and genetic variance shown was the average. The SE was smaller than the figure symbols and is not shown.

An ANOVA was conducted within each combination of *N*_e_ and *h*^2^ (Supplementary Table 2). From ANOVA, SelectSP, and nGP significantly affected GS accuracy in all scenarios (Supplementary Table 2). With the scenario of randomly selecting SPs, GS accuracy was always slightly higher than when selecting the top 10% SPs (Fig. 4). NumCross and CycleTime both had significant effects on GS accuracy except given the scenario when *N*_e_ = 600 and *h*^2^ = 0.5, and the scenario when *N*_e_ = 60 and *h*^2^ = 0.2, respectively (Supplementary Table 2). We also observed a few significant interactions between factors on GS accuracy (Fig. 4; Supplementary Table 2). The GS accuracy overtime across all scenarios slightly increased after 10 years.

## Discussion

Simulation is a useful tool for guiding researchers in decision-making especially for young breeding programs (Zenger *et al.* 2019; Muleta *et al.* 2019). We simulated different breeding schemes, each overcoming a major obstacle we have encountered in 2 seasons of kelp breeding. Assessing which scheme generated the highest genetic gain will allow us to prioritize research directions and derive the most benefit from a limited research budget. Given that aquaculture practice is different from the cultivation of land crops and animals, there are challenges associated with our proposed methods as discussed below.

The simulation revealed a robust result that when other factors remain the same, the highest genetic gain can be achieved by exerting selection on SPs phenotypically rather than randomly (SelectSP, overcoming *Obstacle 3*), and then by reducing the time for obtaining sufficient GP biomass such that a 1-year cycle is enabled compared with our current 2-year cycle (CycleTime, overcoming *Obstacle 1*). Increasing the number of viable GPs we obtain per parental SP (nGP, overcoming *Obstacle 4*) also delivered significant gain, while somewhat surprisingly, phenotyping more SP plots (NumCross, overcoming *Obstacle 2*) did not. These conclusions were not affected by the founder population effective population size or the trait heritability. Thus, the clear direction to prioritize breeding enhancement is to improve our ability to induce SP spore release and to modify GP culture to accelerate growth. In addition, we should experiment with the amount of GP biomass needed to make sufficient SP progeny. We may not need a full 1-m length of plot for evaluation.

It can be challenging to induce top performing SPs to release spores (SelectSP, *Obstacle 3*). Ideally, we aim to select crosses in the top 10% for performance and then artificially induce sporulation in the laboratory. This has proved successful on a small scale if desirable SPs are identified within a day of harvest. Yet compared with the large number of SPs plots we currently have, overcoming *Obstacle 3* requires greater investment in labor to identify and separate candidate SPs and investment in additional culture space to accommodate the induction process. Practically, we have also observed that not all SPs could generate GPs in the laboratory, which could be due to sterility related genes, laboratory environmental conditions, or both. Hence, understanding the mechanisms causing sterile kelp would be beneficial in addressing this obstacle too.

Our second-best option is to accelerate GP growth by overcoming *Obstacle 1* (CycleTime), which could also be the hardest task. In brief, it takes 4–8 weeks to induce immature SPs to full maturity and release meiospores under artificial conditions in the laboratory (Pang and Lüning 2004; Flavin *et al.* 2013; Redmond *et al.* 2014). Once meiospores are released, flow-cytometry techniques can be implemented to isolate single-cell GPs into 96-well plates. A second isolation is performed approximately 2–4 months later when GPs develop into tufts large enough (>100 µm), to be sexed and moved to individual Petri dishes for regular periodic filament fragmentation. Once sufficient uniclonal biomass is achieved (∼10 mg to cover 1 m plots), which can take up to another 4 months, crosses are made by mixing female and male GPs at a 2:1 ratio (Umanzor *et al.* 2021; Fig. 1). Outplanting at sea occurs 4–6 weeks following SP attachment onto the seed string (Flavin *et al.* 2013; Redmond *et al.* 2014). Overall, this process of uniclonal GP isolation, growth and crossing is effective but typically requires 12 months, in contrast to the 6 months available between optimal kelp harvesting (end of May to early June) to crossing and outplanting (November to December).

Generally, GP growth is limited by the natural biological programming of cell division and a propensity to self-shade in its puff-ball growth form. One may argue that the kelp breeding cycle time is determined by this limitation and shortening the cycle time might not be feasible. From our observation, however, some GPs grow faster than others and selecting for GP growth-related traits could be incorporated in the breeding program. There are several possible means of accelerating GP growth including optimizing lighting, nutrient, carbon dioxide, and temperature regimes, as well as novel biomass fragmentation protocols. It might also be possible to optimize GP biomass development by transferring them earlier to plates with bigger wells (i.e. from 96- to 24-well plates) that would allow better light penetration. To test if a 1-year cycle time is feasible in our current breeding program, we have also experimented with using a minimum amount of biomass to make crosses and generate at least a single SP blade. The function of this blade would not be for evaluation of SP performance, but for recombining the best GPs in the hope of getting improved recombinants. This approach would generate phenotypic data on the individual SP blade but not on biomass per meter of line, which is a plot-level trait. Hence this procedure would not be a full representation of the 1-year per cycle scheme we simulated here.

Another possibility that is used in forage breeding (Resende *et al.* 2013) would be to evaluate segregating plots, in our case created from crossing multiple female GPs from 1 SP with multiple male GPs from another SP. The between-plot variance for such mixed plots would be less than that for the uniform SPs plots. However, maintaining multiple individual GPs only until they can be sexed and cocultured together would reduce labor. Such mixed plots would generate sufficient biomass more quickly to facilitate 1-year breeding cycles.

Overcoming *Obstacle 2* (nGP) by increasing the number of GPs per parental SP can potentially be done easily. A simple approach would be to increase the number of plates automatically sorted by flow cytometry per parental SP, which would increase the number of GPs in the GS step, allowing higher selection intensity. Nonetheless, this would result in increasing the number of cultures to maintain in the laboratory, which leads to more labor and cost. The use of flow cytometry sorting expedites the initial isolation process, but the parameters that determine the survival of spores are not well understood. The condition of sorus tissue prior to spore release and sorting likely influences spore viability. Percentage viability varied across samples presumably because of differences in sorus tissue condition and handling prior to sorting (Augyte *et al.* 2020). An issue that should be investigated is whether the selection pressure caused by flow cytometry mortality has pleiotropic effects that might negatively affect SP growth or reproduction. If not, the mortality should generate its own natural selection response that will eventually mitigate this obstacle.

Increasing the number of plots (from 400 to 1,000, NumCross) could be accomplished without new research, but would be costly since it would require more GP grow-out space and labor. This change generated only a small increase in the rate of genetic gain. An important benefit to increasing the number of SPs being phenotyped, however, was that it maintained genetic diversity and slowed down the decrease of genetic variance (Figs. 3 and 4). The proportion of GPs selected out of the SPs was the same regardless of testing 400 or 1,000 plots, hence increasing the number of plots did not change the selection differential. It did, however, affect the training population size of GS models when selecting new generations of GPs. Larger training population size usually contributes to increased GS accuracy (Poland *et al.* 2012; Huang *et al.* 2016). In this case, the increased phenotypic data led to an improved genomic prediction model and its ability to distinguish among-family vs within-family effects. That ability can decrease the coselection of relatives leading to greater maintenance of genetic variation (Jannink *et al.* 2010). Interestingly, every intervention that led to greater genetic gain also led to greater loss of genetic variance for all changes in practice (Selection on SP, Cycle Time, nGP per parental SP), *except* increasing the number of phenotyped plots (NumCross) which both increased gain and decreased variance lost (Table 2; Supplementary Fig. 1). We also observed in some cases that the principal effect of increasing the number of plots was to cause greater variance to be retained, without increasing the gain from selection substantially (in Supplementary Fig. 1 the gray lines were close to vertical). Hence, it seems likely that this intervention would benefit our breeding program over the long term.

While in this discussion we have treated heritability as fixed, that is not strictly true. Heritability might be increased if we could improve our planting technique to ensure that plots are more uniformly covered by SPs, so that we obtain successful and uniform growth of SPs in the field. Not surprisingly, higher heritability leads to greater final gain (Figs. 2 and 3). The decreasing trend of genetic variance led to a relationship where higher final genetic gain coincided with lower genetic variance. It is important to maintain the diversity while we improve the progeny performance (Heffner *et al.* 2009; Lin *et al.* 2016). It is worth noting that environmental effects, such as water temperature, light, etc., play an important role in kelp growth. In our case, we included 2 years of data from a single farm location. As we move forward, data from more years and multiple locations will become available and should be added to update the simulation model to generate practical research suggestions.

Overall, selection causes variance decreases both because of the Bulmer effect and because high fitness ancestors contribute disproportionately to descendants. In our simulations, genetic variance dropped substantially (Fig. 3; Supplementary Fig. 1). This drop is often observed in simulations that assume additive gene action (e.g. Muir 2007; Jannink 2010). With the 1-year per cycle scheme, the population went through 2 times as many selection events as with the 2-year scheme, leading to a greater decrease in genetic variation over the 10 years (Fig. 3; Supplementary Table 1). For all combinations of other factors, there was a higher final genetic variance when nGP was 24 than when it was 96. The increased selection intensity from this intervention caused a greater variance decrease than for any other intervention. The only intervention that caused increased final genetic variance was evaluating more SP plots per year (NumCross = 1,000 vs 400; Supplementary Fig. 1). In this case, increasing the number of plots caused increased effective population size and thus greater maintenance of variance. These effects are also depicted in Supplementary Fig. 1. This increase of the number of SP plots being evaluated also caused an increase of genomic prediction accuracy, which has also been reported to maintain genetic variance (Fig. 4; Supplementary Table 2; Jannink *et al.* 2010). Reducing the CycleTime from 2 years to 1 year also increased GS accuracy (Fig. 4; Supplementary Table 2). Applying random selection scheme at the SP stage also always generated slightly higher GS accuracy for GPs than selecting SPs phenotypically (Fig. 4). This effect was likely due to randomly selected SPs retaining greater variation than phenotypically selected SPs. The GS accuracy at the GP stage steadily increased over breeding cycles. The increased training population size is likely the main reason since each generation added plots to the training population.

In summary, our short-term gain improvement would be achieved if we could improve our ability to induce spores to generate GPs from top selected SP plots on farm, reduce the amount of time it takes for GPs to be ready for crossing, and increase the number GPs generated per SP. These means we could exert a positive selection on SPs and reduce the breeding cycle time. Form a long-term perspective, however, it is beneficial to increase the number of plots being tested on farm, which helps maintain the genetic diversity. The creation of new variation such as through collecting wild samples should also be an ongoing effort in the program. Overall, the robustness of these simulation findings should give us confidence in the research directions they suggest. We believe that these priorities will help accelerate genetic gain in breeding programs and therefore increase the value of kelp farming in the United States and globally.

## Data availability

Supplementary Table 1 is available as a zip file. Founder genotypic data and trait heritabilitis for kelp, simulation codes in R are available on github: https://github.com/MaoHuang2020/SimulationKelp/tree/master/For_G3. All data necessary for confirming the conclusions of the manuscript are present within the article, figures, and tables.

Supplemental material is available at *G3* online.

## Supplementary Material

jkac003_Supplemental_Tabes_1_and_2

jkac003_Supplemental_Figure_1
