# Correction to: Simulation of sugar kelp (*Saccharina latissima*) breeding guided by practices to accelerate genetic gains

**DOI:** 10.1093/g3journal/jkac186

**Published:** 2022-08-27

This is a correction to: Mao Huang, Kelly R. Robbins, Yaoguang Li, Schery Umanzor, Michael Marty-Rivera, David Bailey, Charles Yarish, Scott Lindell, Jean-Luc Jannink, Simulation of sugar kelp (*Saccharina latissima*) breeding guided by practices to accelerate genetic gains, G3 Genes|Genomes|Genetics, Volume 12, Issue 3, March 2022, https://doi.org/10.1093/g3journal/jkac003

In the article by M. Huang et al., entitled “Simulation of sugar kelp (*Saccharina latissima*) breeding guided by practices to accelerate genetic gains,” Figure 4 was mistakenly placed where Figure 1b should have been. This has now been corrected and on page 3, the content of Figure 1b is shown.

