# Supplementary material for: Simulation of sugar kelp (Saccharina latissima) breeding guided by practices to accelerate genetic gains
Source: G3 (Bethesda). 2022 Jan 19;12(3):jkac003. doi: 10.1093/g3journal/jkac003 (PMC8895986; doi:10.1093/g3journal/jkac003)
Supplement: jkac003_Supplemental_Tabes_1_and_2 [file jkac003_supplemental_tabes_1_and_2.docx]

Supplemental Tables

Supplemental Table 1. ANOVA on total Genetic Variance split by founder effective population size (*N_e_*) and heritability (*h^2^*).

a. *N_e_* = 60, *h^2^* = 0.5

|  | Df | Sum Sq | Mean Sq | *F* | *P-value* |
| --- | --- | --- | --- | --- | --- |
| SelectSP^†^ | 1 | 0.1 | 0.1 | 15.9 | 0.000*** |
| NumCross | 1 | 0.0 | 0.0 | 4.4 | 0.039* |
| CycleTime | 1 | 0.1 | 0.1 | 11.2 | 0.001** |
| nGP | 1 | 0.2 | 0.2 | 19.8 | 0.000*** |
| SelectSP:NumCross | 1 | 0.0 | 0.0 | 0.0 | 0.908 |
| SelectSP:CycleTime | 1 | 0.0 | 0.0 | 0.4 | 0.551 |
| SelectSP:nGP | 1 | 0.0 | 0.0 | 0.4 | 0.549 |
| NumCross:CycleTime | 1 | 0.0 | 0.0 | 0.4 | 0.515 |
| NumCross:nGP | 1 | 0.0 | 0.0 | 0.2 | 0.639 |
| CycleTime:nGP | 1 | 0.0 | 0.0 | 1.1 | 0.297 |
| Residuals | 101 | 0.8 | 0.0 |  |  |

b. *N_e_* = 600, *h^2^* = 0.5

|  | Df | Sum Sq | Mean Sq | *F* | *P-value* |
| --- | --- | --- | --- | --- | --- |
| SelectSP† | 1 | 0.2 | 0.2 | 22.4 | 0.000*** |
| NumCross | 1 | 0.1 | 0.1 | 10.1 | 0.002** |
| CycleTime | 1 | 0.1 | 0.1 | 12.8 | 0.001*** |
| nGP | 1 | 0.2 | 0.2 | 30.9 | 0.000*** |
| SelectSP:NumCross | 1 | 0.0 | 0.0 | 0.0 | 0.852 |
| SelectSP:CycleTime | 1 | 0.0 | 0.0 | 0.1 | 0.752 |
| SelectSP:nGP | 1 | 0.0 | 0.0 | 0.6 | 0.455 |
| NumCross:CycleTime | 1 | 0.0 | 0.0 | 0.5 | 0.470 |
| NumCross:nGP | 1 | 0.0 | 0.0 | 1.1 | 0.287 |
| CycleTime:nGP | 1 | 0.0 | 0.0 | 2.1 | 0.154 |
| Residuals | 101 | 0.8 | 0.0 |  |  |

c. *N_e_* = 60, *h^2^* = 0.2

|  | Df | Sum Sq | Mean Sq | *F* | *P-value* |
| --- | --- | --- | --- | --- | --- |
| SelectSP† | 1 | 0.0 | 0.0 | 4.6 | 0.034* |
| NumCross | 1 | 0.1 | 0.1 | 7.6 | 0.007** |
| CycleTime | 1 | 0.1 | 0.1 | 14.7 | 0.000*** |
| nGP | 1 | 0.2 | 0.2 | 27.7 | 0.000*** |
| SelectSP:NumCross | 1 | 0.0 | 0.0 | 0.0 | 0.992 |
| SelectSP:CycleTime | 1 | 0.0 | 0.0 | 0.1 | 0.702 |
| SelectSP:nGP | 1 | 0.0 | 0.0 | 0.0 | 0.843 |
| NumCross:CycleTime | 1 | 0.0 | 0.0 | 0.4 | 0.541 |
| NumCross:nGP | 1 | 0.0 | 0.0 | 0.8 | 0.378 |
| CycleTime:nGP | 1 | 0.0 | 0.0 | 1.8 | 0.182 |
| Residuals | 101 | 0.8 | 0.0 |  |  |

d. *N_e_* = 600, *h^2^* = 0.2

|  | Df | Sum Sq | Mean Sq | *F* | *P-value* |
| --- | --- | --- | --- | --- | --- |
| SelectSP† | 1 | 0.0 | 0.0 | 4.3 | 0.041* |
| NumCross | 1 | 0.1 | 0.1 | 12.2 | 0.001*** |
| CycleTime | 1 | 0.2 | 0.2 | 16.4 | 0.000*** |
| nGP | 1 | 0.3 | 0.3 | 33.9 | 0.000*** |
| SelectSP:NumCross | 1 | 0.0 | 0.0 | 0.0 | 0.957 |
| SelectSP:CycleTime | 1 | 0.0 | 0.0 | 0.0 | 0.994 |
| SelectSP:nGP | 1 | 0.0 | 0.0 | 0.1 | 0.773 |
| NumCross:CycleTime | 1 | 0.0 | 0.0 | 1.0 | 0.329 |
| NumCross:nGP | 1 | 0.0 | 0.0 | 1.2 | 0.268 |
| CycleTime:nGP | 1 | 0.0 | 0.0 | 2.5 | 0.120 |
| Residuals | 101 | 1.0 | 0.0 |  |  |

* P<0.05,** P<0.001, *** P<0.0001

^†^ SelectSP: Selection among SP based on phenotype or at random. NumCross: Common garden of 400 versus 1000 field plots. CycleTime: 1-year versus 2-year cycle. nGP: number of GPs obtained per parental SP of 24 or 96.

Supplemental Table 2. ANOVA on Genomic Selection accuracy split by founder effective population size (*N_e_*) and heritability (*h^2^*).

a. *N_e_* = 60, *h^2^* = 0.5

|  | Df | Sum Sq | Mean Sq | *F* | *P-value* |
| --- | --- | --- | --- | --- | --- |
| SelectSP^†^ | 1 | 0.1 | 0.1 | 159.3 | < 2.20E-16 *** |
| NumCross | 1 | 0.1 | 0.1 | 328.3 | < 2.20E-16 *** |
| CycleTime | 1 | 0.0 | 0.0 | 7.4 | 0.008 ** |
| nGP | 1 | 0.0 | 0.0 | 10.8 | 0.001 ** |
| SelectSP:NumCross | 1 | 0.0 | 0.0 | 12.0 | 0.001 *** |
| SelectSP:CycleTime | 1 | 0.0 | 0.0 | 0.1 | 0.726 |
| SelectSP:nGP | 1 | 0.0 | 0.0 | 0.1 | 0.734 |
| NumCross:CycleTime | 1 | 0.0 | 0.0 | 10.2 | 0.002 ** |
| NumCross:nGP | 1 | 0.0 | 0.0 | 3.2 | 0.078 |
| CycleTime:nGP | 1 | 0.0 | 0.0 | 0.3 | 0.574 |
| Residuals | 77 | 0.0 | 0.0 |  |  |

b. *N_e_* = 600, *h^2^* = 0.5

|  | Df | Sum Sq | Mean Sq | *F* | *P-value* |
| --- | --- | --- | --- | --- | --- |
| SelectSP† | 1 | 0.2 | 0.2 | 130.4 | < 2.20E-16 *** |
| NumCross | 1 | 0.0 | 0.0 | 0.0 | 0.981 |
| CycleTime | 1 | 0.0 | 0.0 | 18.9 | 0.000 *** |
| nGP | 1 | 0.0 | 0.0 | 23.4 | 0.000 *** |
| SelectSP:NumCross | 1 | 0.0 | 0.0 | 1.2 | 0.282 |
| SelectSP:CycleTime | 1 | 0.0 | 0.0 | 0.1 | 0.745 |
| SelectSP:nGP | 1 | 0.0 | 0.0 | 6.6 | 0.012 * |
| NumCross:CycleTime | 1 | 0.0 | 0.0 | 0.3 | 0.587 |
| NumCross:nGP | 1 | 0.0 | 0.0 | 2.9 | 0.094 |
| CycleTime:nGP | 1 | 0.0 | 0.0 | 0.0 | 0.912 |
| Residuals | 77 | 0.1 | 0.0 |  |  |

c. *N_e_* = 60, *h^2^* = 0.2

|  | Df | Sum Sq | Mean Sq | *F* | *P-value* |
| --- | --- | --- | --- | --- | --- |
| SelectSP† | 1 | 0.0 | 0.0 | 45.3 | 0.000 *** |
| NumCross | 1 | 0.1 | 0.1 | 207.4 | < 2.20E-16 *** |
| CycleTime | 1 | 0.0 | 0.0 | 0.5 | 0.497 |
| nGP | 1 | 0.0 | 0.0 | 4.2 | 0.044 * |
| SelectSP:NumCross | 1 | 0.0 | 0.0 | 1.8 | 0.182 |
| SelectSP:CycleTime | 1 | 0.0 | 0.0 | 0.2 | 0.639 |
| SelectSP:nGP | 1 | 0.0 | 0.0 | 0.1 | 0.800 |
| NumCross:CycleTime | 1 | 0.0 | 0.0 | 7.5 | 0.008 ** |
| NumCross:nGP | 1 | 0.0 | 0.0 | 0.4 | 0.547 |
| CycleTime:nGP | 1 | 0.0 | 0.0 | 0.1 | 0.715 |
| Residuals | 77 | 0.1 | 0.0 |  |  |

d. *N_e_* = 600, *h^2^* = 0.2

|  | Df | Sum Sq | Mean Sq | *F* | *P-value* |
| --- | --- | --- | --- | --- | --- |
| SelectSP† | 1 | 0.1 | 0.1 | 55.0 | 0.000 *** |
| NumCross | 1 | 0.0 | 0.0 | 9.4 | 0.003 ** |
| CycleTime | 1 | 0.0 | 0.0 | 14.7 | 0.000 *** |
| nGP | 1 | 0.0 | 0.0 | 9.3 | 0.003 ** |
| SelectSP:NumCross | 1 | 0.0 | 0.0 | 7.6 | 0.007 ** |
| SelectSP:CycleTime | 1 | 0.0 | 0.0 | 0.0 | 0.920 |
| SelectSP:nGP | 1 | 0.0 | 0.0 | 0.0 | 0.935 |
| NumCross:CycleTime | 1 | 0.0 | 0.0 | 1.1 | 0.296 |
| NumCross:nGP | 1 | 0.0 | 0.0 | 6.8 | 0.011 * |
| CycleTime:nGP | 1 | 0.0 | 0.0 | 0.7 | 0.420 |
| Residuals | 77 | 0.1 | 0.0 |  |  |

* P<0.05,** P<0.001, *** P<0.0001

^†^ SelectSP: Selection among SP based on phenotype or at random. NumCross: Common garden of 400 versus 1000 field plots. CycleTime: 1-year versus 2-year cycle. nGP: number of GPs obtained per parental SP of 24 or 96.
