# Supplementary material for: Simulation of sugar kelp (Saccharina latissima) breeding guided by practices to accelerate genetic gains
Source: G3 (Bethesda). 2022 Jan 19;12(3):jkac003. doi: 10.1093/g3journal/jkac003 (PMC8895986; doi:10.1093/g3journal/jkac003)

Supplemental Figure 1

Four views of the simulation results on the final genetic variance and genetic mean. Each view presents the same scatterplot, with each point representing the mean outcome of 20 simulations of one scheme. Each view shows a different obstacle to overcome, with the color of the point determined by the current practice (black) or the improved practice (red). Gray lines connect simulation schemes that are identical except for this change in practice.

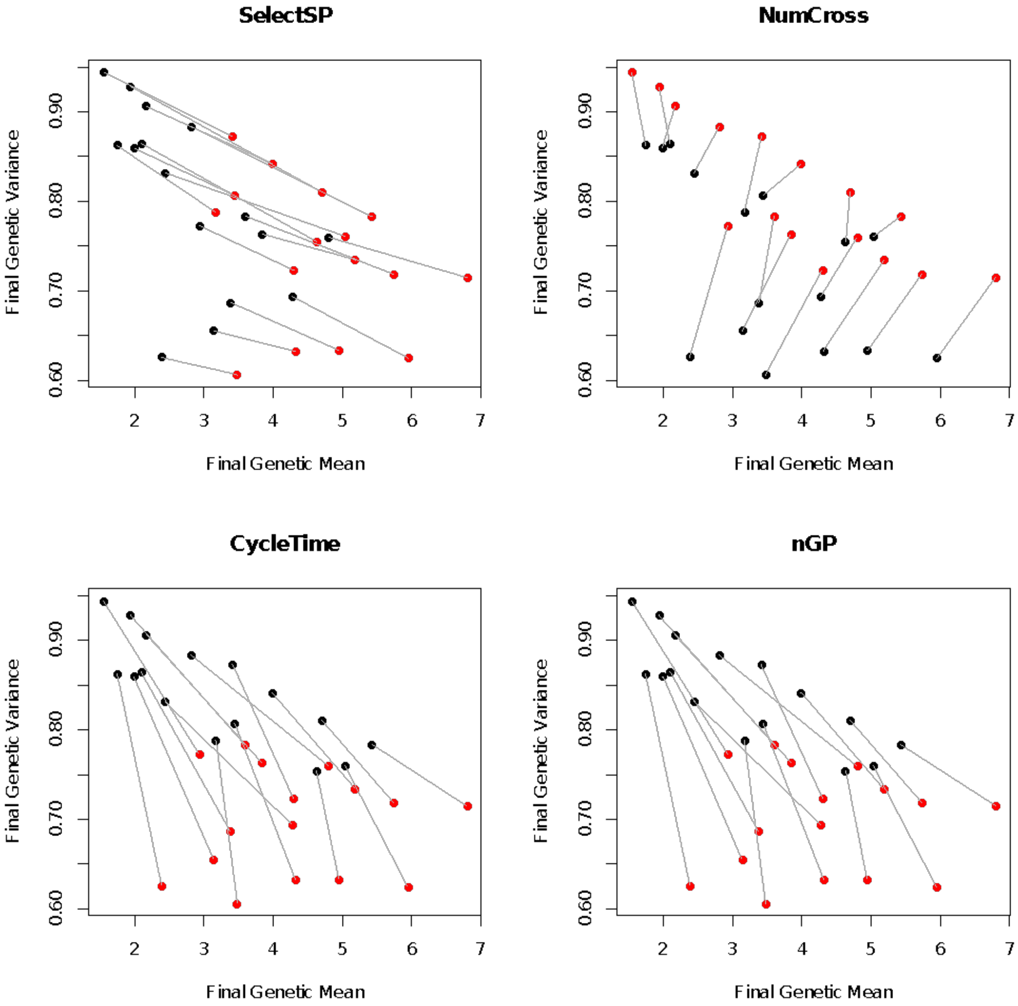

Supplement: jkac003_Supplemental_Figure_1 [file jkac003_supplemental_figure_1.pdf]
